# Effect of Acetazolamide on Obesity-Induced Glomerular Hyperfiltration: A Randomized Controlled Trial

**DOI:** 10.1371/journal.pone.0137163

**Published:** 2015-09-14

**Authors:** Boris Zingerman, Michal Herman-Edelstein, Arie Erman, Sarit Bar Sheshet Itach, Yaacov Ori, Benaya Rozen-Zvi, Uzi Gafter, Avry Chagnac

**Affiliations:** 1 Department of Nephrology & Hypertension, Rabin Medical Center, Petah Tikva, Israel; 2 Sackler School of Medicine, Tel Aviv University, Tel Aviv, Israel; University Medical Center Utrecht, NETHERLANDS

## Abstract

**Aims:**

Obesity is an important risk factor for the development of chronic kidney disease. One of the major factors involved in the pathogenesis of obesity-associated kidney disease is glomerular hyperfiltration. Increasing salt-delivery to the macula densa is expected to decrease glomerular filtration rate (GFR) by activating tubuloglomerular feedback. Acetazolamide, a carbonic anhydrase inhibitor which inhibits salt reabsorption in the proximal tubule, increases distal salt delivery. Its effects on obesity-related glomerular hyperfiltration have not previously been studied. The aim of this investigation was to evaluate whether administration of acetazolamide to obese non diabetic subjects reduces glomerular hyperfiltration.

**Materials and Methods:**

The study was performed using a randomized double-blind crossover design. Obese non-diabetic men with glomerular hyperfiltration were randomized to receive intravenously either acetazolamide or furosemide at equipotent doses. Twelve subjects received the allocated medications. Two weeks later, the same subjects received the drug which they had not received during the first study. Inulin clearance, p-aminohippuric acid clearance and fractional lithium excretion were measured before and after medications administration. The primary end point was a decrease in GFR, measured as inulin clearance.

**Results:**

GFR decreased by 21% following acetazolamide and did not decrease following furosemide. Renal vascular resistance increased by 12% following acetazolamide, while it remained unchanged following furosemide administration. Natriuresis increased similarly following acetazolamide and furosemide administration. Sodium balance was similar in both groups.

**Conclusions:**

Intravenous acetazolamide decreased GFR in obese non-diabetic men with glomerular hyperfiltration. Furosemide, administered at equipotent dose, did not affect GFR, suggesting that acetazolamide reduced glomerular hyperfiltration by activating tubuloglomerular feedback.

**Trial Registration:**

ClinicalTrials.gov NCT01146288

## Introduction

Obesity is an independent risk factor for chronic kidney disease[1]. Obesity-related glomerulopathy is a well-defined entity, characterized by glomerulomegaly with or without focal segmental glomerulosclerosis[2]. In addition, increased adiposity also accelerates the progression of kidney diseases that are not primarily related to obesity, such as IgA nephropathy[3,4], reduced renal mass[5] and possibly renal transplant nephropathy[6]. Obesity and overweight are associated with increased GFR, renal plasma flow and/or filtration fraction[7–15], with central body fat distribution being a more powerful predictor of hyperfiltration than body mass index (BMI)[16]. The improvement of these renal hemodynamic abnormalities following weight loss[17] supports a cause-and-effect relationship between adiposity and glomerular hyperfiltration.

One of the factors involved in the pathogenesis of obesity-associated kidney disease is glomerular hyperfiltration[18–25], which is associated with afferent arteriolar vasodilatation and increased glomerular pressure[11]. Treatment of chronic kidney disease in the obese subject is oriented, among other things, toward decreasing glomerular pressure by antihypertensive treatment, inhibition of the renin angiotensin system and weight loss. The latter decreases arterial hypertension and restores renal hemodynamics toward normal, reducing renal plasma flow, GFR and filtration fraction[7,17]. However, this treatment has its limitations, weight loss being rarely maintained in the long term in obese subjects.

Activation of tubuloglomerular feedback by increased sodium distal delivery and the consequent decrease in GFR is a yet unexplored way of modulating glomerular hyperfiltration in obesity. Tucker et al[26] showed that administration of a carbonic anhydrase inhibitor in normal rats leads to a decrease in single-nephron GFR by inhibiting proximal tubular reabsorption, increasing salt delivery to the macula densa and thus activating tubuloglomerular feedback. This GFR reducing effect of carbonic anhydrase inhibition has also been demonstrated in normal humans[27–33] and in diabetic subjects[31,34]. However, these studies did not use a control group treated with an equipotent natriuretic agent. Recently, Cherney et al showed, in lean subjects with type 1 diabetes mellitus and glomerular hyperfiltration, that increasing solute delivery to the macula densa by inhibition of proximal tubular reabsorption attenuates hyperfiltration[35]. The effects of inhibition of proximal tubular reabsorption have not been previously studied in an obese non-diabetic population.

The present study was designed to test the hypothesis that administration of acetazolamide, a carbonic anhydrase inhibitor, to obese subjects with glomerular hyperfiltration reduces hyperfiltration. The effects of acetazolamide on renal hemodynamics were compared to those of furosemide, a loop diuretic that does not activate tubuloglomerular feedback[28,30,36–38]. We showed that intravenous acetazolamide decreases GFR in non-diabetic obese men with glomerular hyperfiltration, while furosemide, administered at equipotent natriuretic dose, does not affect GFR.

## Materials and Methods

### Ethics Statement

The protocol of this study was approved by the Institutional Review Board of the Rabin Medical Center. Informed consent was signed by the participants.

### Study design

Randomized double-blind crossover controlled.

### Participants

Eligible participants were men aged 18 to 55 years old with a BMI above 30 kg/m^2^ and a creatinine clearance above 130 ml/min. Creatinine clearance was measured using a 24-hour urine collection and a serum blood test for creatinine. Exclusion criteria were any of the following conditions: pharmacologic treatment for diabetes mellitus, hypertension and cardiac disease; history of kidney disease, heart failure and chronic obstructive lung disease; therapy with corticosteroid, antiepileptic and non steroidal anti inflammatory medications; known allergy to furosemide, acetazolamide, inulin and amino-hippurate.

### Interventions

Enrolled patients underwent two renal function studies, one before and after intravenous administration of acetazolamide and the other before and after intravenous administration of furosemide, which was used as control. The order of administration was randomized. The effects of acetazolamide were compared to those of furosemide, since both medications are natriuretic, but the latter increases natriuresis with no activation of the tubuloglomerular feedback[28,30,36–38]. Acetazolamide was administered at a dose of 5 mg/kg BW. Preliminary studies performed in our lab showed that a furosemide dose of 2 mg was equipotent to that of acetazolamide dose, as far as natriuresis was concerned.

A 24-hour urine collection was performed before the two renal function studies. Subjects received 300 mg of lithium carbonate at 22.00 the day before the tests. They were instructed to drink 250 ml of water at bedtime and at 07.00 am. Renal function tests were started at 08.00 am. after a 10-hour fast, excepting the water drink. Intravenous catheters were placed in each upper limb for infusion of clearance markers and blood sampling. After blood sampling for urea, creatinine, proteins, glucose, electrolytes, blood gases, HbA1c and complete blood count, a priming dose of inulin (40 mg/kg) and p-aminohippuric acid (4 mg/kg) was intravenously injected followed by ingestion of 10 ml/kg of water. Thereafter, inulin and p-aminohippuric acid were infused continuously at a rate of 33 and 17 mg/min, respectively. After each voiding, participants drank an amount of water equal to the amount of urine voided. After the first 60 minutes, 4 accurately timed urine collections of 30 minutes each were obtained by spontaneous voiding. Peripheral venous blood was drawn to bracket each urine collection. Arterial pressure was measured by a trained observer, at the end of each urine collection in the supine position, using an electronic oscillometric blood pressure measuring device (Datascope, Accutorr). The cuff was appropriately sized to the diameter of the arm and the arm positioned at the heart level. Each measurement was the mean of 3 readings. After the first 4 timed urine collections, participants received either intravenous acetazolamide at a dose of 5 mg/kg within 5 min or intravenous furosemide at a dose of 2 mg within 5 min. The drugs were injected after dilution in 0.9% sodium chloride as a 20 ml solution. Following the injection, a 0.9% sodium chloride solution was administered intravenously at a rate of 130 ml/hr and two 30-minute urine collections were performed. The second study was performed 14 (13–22) days after the first study, using the drug that had not been administered during the first study. Blood sampled before and after each urine collection was assayed for albumin, protein, sodium, inulin and p-aminohippuric acid. Each of the urine collections was assayed for sodium, inulin and p-aminohippuric acid. Lithium was measured in 2 urine collections preceding diuretic administration and in urine samples collected 30 and 60 min following diuretic administration, as well as in blood samples bracketing these collections. Venous blood gases were measured in blood samples drawn at the end of the two last baseline urine collections and in samples drawn 30 and 60 min following diuretic administration.

**Fig 1:**
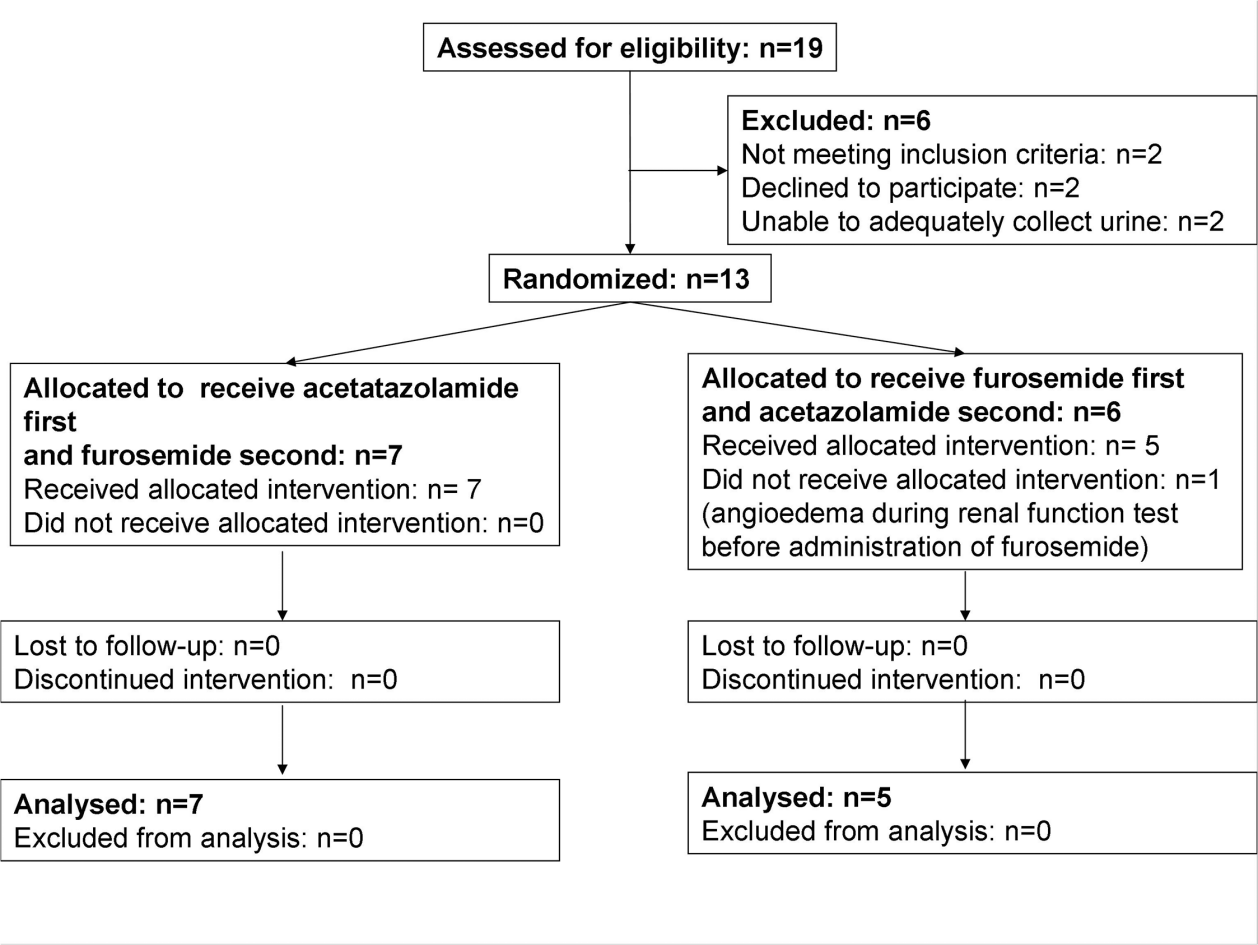
Investigation's flowchart.

The study was performed using a randomized double-blind crossover comparative design. The order of administration was randomized. Fig 1 shows the investigation's flowchart. Nineteen subjects were assessed for eligibility. Twelve subjects received the allocated treatment. Data obtained from the studies of all 12 subjects were analyzed.

### Randomization and masking

Participants were assigned to receive either acetazolamide or furosemide during the first renal function test using a simple randomization procedure (computerized random numbers) prepared by a pharmacist not otherwise involved in the study. Medications were prepared by a nurse not otherwise involved in the study and injected by one of the authors (BZ) who was unaware of the medication administered. The participants, the coauthors and everyone involved in the laboratory procedures and data analysis were blinded to the medication administered until completion of data analysis.

### Outcome

The primary endpoint of this study was a change in GFR following diuretic administration.

### Date range for participants' recruitment

2011.01.31 to 2012.08.27

### Laboratory procedures

Plasma and urinary concentrations of inulin and p-aminohippuric acid were analyzed by colorimetric methods. Lithium in the serum and urine was measured using the ICP-OES (Inductively Coupled Plasma Optical Emission Spectrometer) method. Urine albumin was measured using chemiluminescence. HbA1c, plasma glucose, sodium, creatinine, albumin, protein and bicarbonate and urine sodium, creatinine and urea nitrogen were measured using standard laboratory methods. HbA1c was measured in 11 out of the 12 obese subjects. The subject with missing HbA1c had a fasting blood glucose level of 96 mg/dl.

### Calculations

GFR and renal plasma flow were calculated as inulin and p-aminohippuric acid sodium clearance, respectively. Baseline GFR and renal plasma flow were calculated from the average value of the four inulin and p-aminohippuric acid sodium clearance measurements performed before diuretic administration. BMI was calculated as: Body weight/Height^2^, body weight being expressed in kg and height expressed in m. The fractional lithium excretion was calculated as: lithium clearance/GFR. Baseline fractional lithium excretion was determined as the average value for two measurements performed before diuretic administration. Renal blood flow was calculated as: Renal plasma flow/(1—Hematocrit), flow being expressed in ml/min and hematocrit as a fraction. Mean arterial pressure was calculated as: (Systolic arterial pressure+2xDiastolic arterial pressure)/3, pressure being expressed in mm Hg. Renal vascular resistance, expressed in mm Hg/(ml/min), was calculated as: Mean arterial pressure/ Renal blood flow. Albumin excretion rate was calculated as the mean value from the two 24-hour urine collections. Protein intake was calculated as[39]: (Urine urea nitrogen + [Body weight x 0.031]) x 6.25, protein intake being expressed in g/d, urine urea nitrogen in g/d and body weight in kg. Sodium balance during renal function studies, i.e. from the time of inulin and p-aminohippuric acid injection to that of the end of the last urine collection, was calculated as: Intravenous sodium intake + Oral sodium intake (as drinking water)–Urinary sodium excretion, expressed in mmol.

### Statistical Analysis

Normally distributed data are expressed as mean±SD, unless otherwise specified. Variables with skewed distribution are expressed as median (range). Analysis was performed according to the intention-to-treat principle, with all subjects who received the allocated medication being included in the analysis. The significance of differences between groups was evaluated by ANOVA with repeated measures. The effect of administration order of the drugs was evaluated for the primary outcome. When ANOVA showed a significant treatment-by-time interaction, a paired t-test was applied. Regression analysis between variables was performed after log-transformation for non-normally distributed variables. Missing data: serum albumin and serum bicarbonate data were unavailable for one subject each. Analysis for these two variables was performed for 11 out of the 12 subjects.

All tests were two-sided. P<0.05 was considered as significant. The analyses were carried using SPSS software version 21.

### Sample size

Sample size calculation was based on the results of a study by Hannedouche et al^31^ on the effects of intravenously administrated acetazolamide on renal hemodynamics in healthy and diabetic subjects. GFR decreased by 14±10% following acetazolamide. The SD of the treatment effect was calculated using data appearing in Fig 1 of this publication. Using these data, we calculated that it would be necessary to include 15 subjects in the present investigation in order to demonstrate a 14% decrease in GFR, assuming an alpha of 0.005, a power of 90% and a drop-out of up to 20% after randomization.

### Interim Analysis

Due to slow enrollment, an interim analysis was performed after 13 subjects had been randomized and 12 had completed the study. This analysis showed that the primary endpoint was reached. Due to this result and the slow enrollment rate, the study was stopped before completion of the randomization of 15 subjects, as initially planned.

## Results

Twelve obese male subjects, aged 36 (32–53) received the allocated medications. Body weight was 122±19 kg and height – 1.78±0.09 m. Body mass index was 38.8±5.7 kg/m^2^ (range: 31.6–49.5) and waist circumference – 124±12 cm (range: 109–141). Serum creatinine was 69.8±6.2 μmol/L. Fasting blood glucose was 5.05 (4.55–6.49) mmol/L and HbA1c – 5.5 (5.3–6.2)%. Creatinine clearance was 177±27 ml/min. Albumin excretion rate was 12.4 (range: 5.9–39.7; lower quartile: 9.8; upper quartile: 21.9) mg/d. Calculated protein intake, assessed during the day preceding acetazolamide and furosemide studies using 24-hour urine collections, was 115±21 and 111±22 g/d respectively (P NS).

Sodium intake during the day preceding acetazolamide and furosemide studies, as assessed by a 24-hour urine collection, was 248 (105–399) and 262 (123–344) mmol, respectively (P = 0.9). During baseline renal function studies, systolic arterial pressure was 122±11 mm Hg before both acetazolamide and furosemide administration; diastolic arterial pressure was 80±11 and 80±10 mm Hg before acetazolamide and furosemide administration, respectively (P = 0.9).

### Renal Hemodynamics

**Fig 2:**
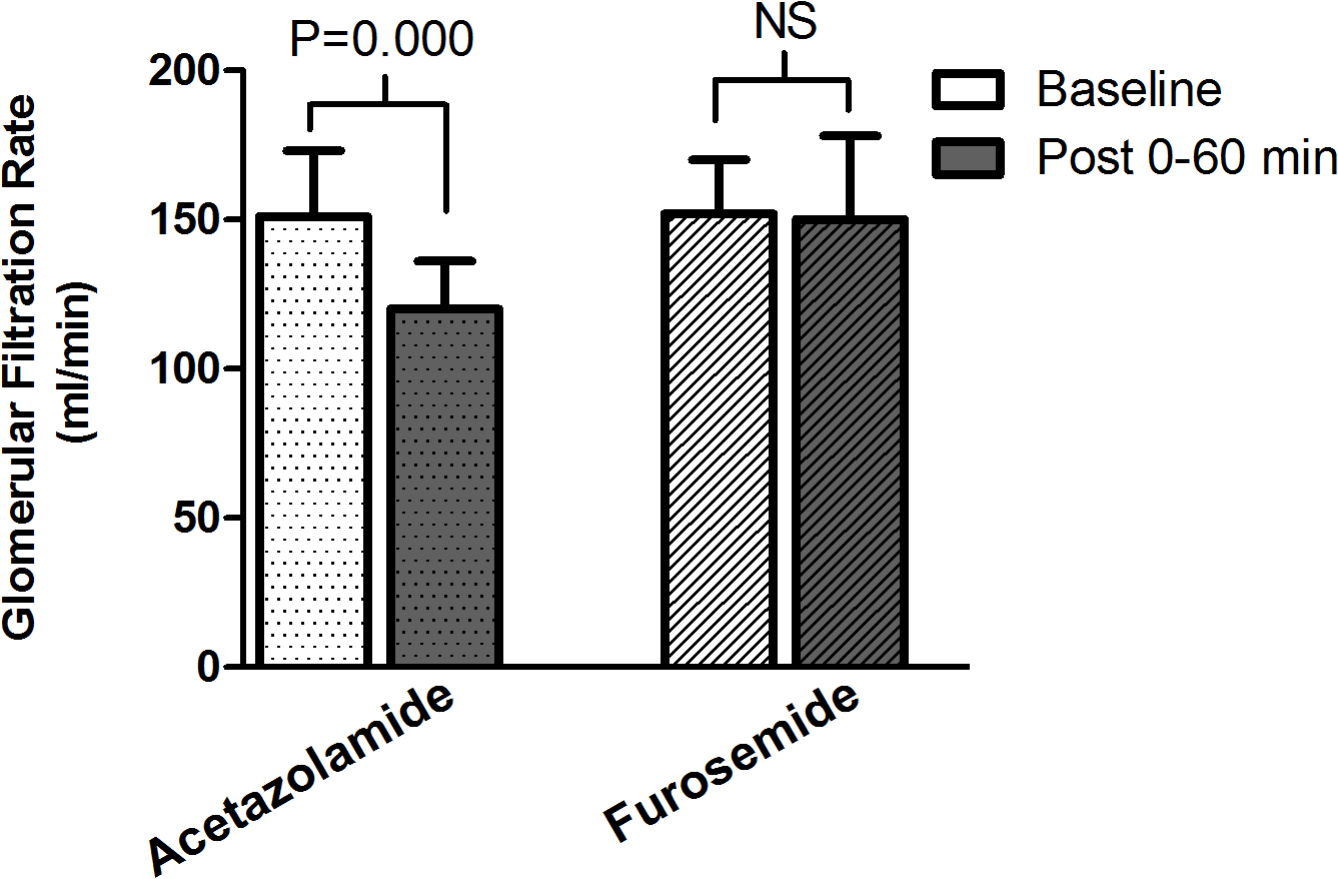
GFR before (Baseline) and after (Post) acetazolamide and furosemide administration. GFR decreased by 21% following acetazolamide and remained unchanged following furosemide.

**Fig 3:**
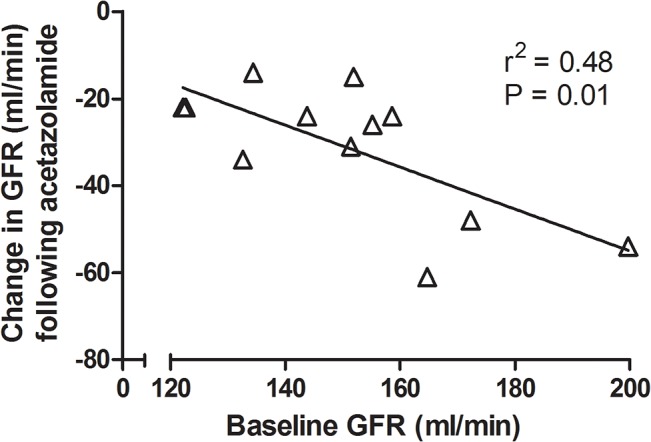
Change in GFR following acetazolamide administration as a function of baseline GFR. Baseline GFR and the change in GFR following acetazolamide were inversely correlated.

Table 1 and Fig 2 show the renal hemodynamic changes occurring after acetazolamide and furosemide administration. Repeated-measures analysis of variance of GFR (Table 1) showed a significant interaction between treatment and time (P = 0.001), i.e. the effect of acetazolamide on GFR differed from that of furosemide. GFR decreased by 21% following acetazolamide (P<0.001), while it remained unchanged following furosemide administration (Fig 2). Order of administration of the study medications did not affect GFR change. Repeated-measures analysis of variance revealed no significant effect of time on RPF. However, the P value from a paired t-test before, as compared to after, acetazolamide was 0.03, suggesting a trend toward significance. Repeated-measures analysis of variance showed a significant effect of time on renal vascular resistance (P = 0.03) with no significant interaction between treatment and time. Renal vascular resistance increased by 12% following acetazolamide (P = 0.01), while it remained unchanged following furosemide administration (P = 0.1). Fig 3 shows that baseline GFR and the change in GFR following acetazolamide were inversely correlated (r = -0.69, P = 0.01), baseline GFR accounting for 48% of the effect of acetazolamide on GFR. Baseline albuminuria and the change in GFR following acetazolamide were not correlated (r = 0.36, P = 0.25).

**Table 1:** Renal Hemodynamics before and after Acetazolamide and Furosemide in Obese Subjects.

	Acetazolamide		Furosemide	
	Pre	Post	Pre	Post
**Glomerular filtration rate (ml/min)**	151±22	120±16*	152±18	150±28
**Renal plasma flow (ml/min)**	735±167	675±168	783±75	775±190
**Renal vascular resistance (mm Hg/[ml/min])**	0.078±0.017	0.088±0.021^†^	0.073±0.010	0.080±0.019

* P = 0.000

† P = 0.015

### Sodium handling

**Fig 4:**
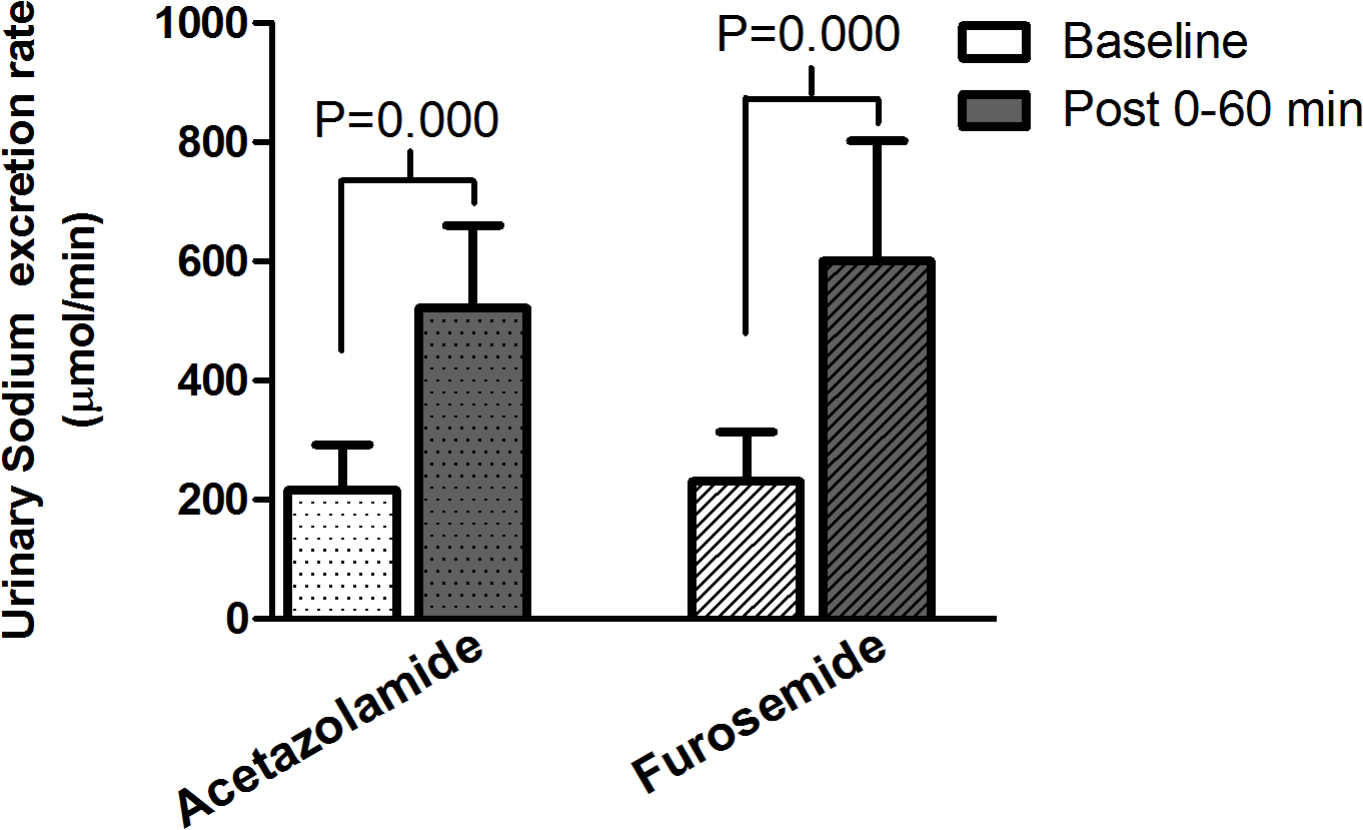
Urinary sodium excretion rate before (Baseline) and after (Post) acetazolamide and furosemide administration. Natriuresis increased similarly following acetazolamide and furosemide administration.

Table 2 and Fig 4 show renal sodium handling before and after acetazolamide and furosemide injection. Serum sodium remained constant during the whole study. Natriuresis was similar during the two baseline renal function studies preceding medications administration. Repeated-measures analysis of variance revealed no significant interaction between treatment and time for natriuresis, while the effect of time was significant, indicating that acetazolamide and furosemide affected sodium excretion. Urinary sodium excretion increased similarly following administration of the 2 medications. Natriuresis increased by 141% following acetazolamide (P<0.001) and by 151% following furosemide injection (P<0.001). Lithium clearance, a marker of sodium reabsorption by the proximal tubule, was similar during the baseline renal function studies preceding drugs administration. It increased 1.7 times more following acetazolamide than following furosemide (30% vs 18%, P<0.03). Fractional lithium excretion, was similar during the baseline renal function studies preceding drugs administration. It increased 2.3 times more following acetazolamide than following furosemide (61% vs 27%, P = 0.002). Sodium balance during renal function studies was similar following acetazolamide and furosemide, -30±20 mmol and -35±26 mmol, respectively (P = 0.35).

**Table 2:** Renal Sodium Handling before and after Acetazolamide and Furosemide in Obese Subjects.

	Acetazolamide		Furosemide	
	Pre	Post	Pre	Post
**Serum sodium (mmol/L)**	138.8±1.5	139.0±1.6	138.7±1.7	138.5±1.9
**Urine sodium excretion rate (μmol/min)**	216±76	521±139*	239±84	600±202*
**Fractional lithium excretion**	0.166±0.056	0.267±0.058*	0.167±0.050	0.212±0.062^‡^
**Lithium clearance (ml/min)**	24.6±8.3	31.9±8.9*	24.8±7.1	29.2±8.7^‡^

* P = 0.000

‡ P = 0.02

Table 3 shows serum protein and arterial pressure during the study. Systolic and diastolic arterial pressures were similar during baseline studies. Systolic arterial pressure remained constant following both acetazolamide and furosemide administration. Diastolic arterial pressure increased by 3 mm Hg following furosemide injection (P = 0.02) and remained constant following acetazolamide administration (P = 0.9). Serum albumin and total protein remained constant before and after acetazolamide and furosemide administration. Plasma bicarbonate was 24.2±1.4 and 24.7±1.4 mEq/L before and after furosemide (P = 0.1). It decreased from 24.2±1.1 to 23.1±1.1 mEq/L following acetazolamide (P = 0.002).

**Table 3:** Serum Protein and Arterial Pressure before and after Acetazolamide and Furosemide in Obese Subjects.

	Acetazolamide		Furosemide	
	Pre	Post	Pre	Post
**Serum albumin**	**46.3±3.1**	**46.1±3.0**	**46.2±2.9**	**45.9±3.2**
**(g/L)**				
**Serum total protein**	**72.3±3.1**	**72.2±3.6**	**71.6±3.9**	**71.4±3.9**
**(g/L)**				
**Systolic arterial pressure (mm Hg)**	**127±11**	**128±10**	**127±11**	**130±10**
**Diastolic arterial pressure (mm Hg)**	**80±11**	**80±11**	**80±10**	**83±10** ^§^

§ P = 0.025

### Adverse effects of allocated medications

Adverse events occurred in 3 subjects following acetazolamide administration: hand paresthesia (two subjects), lip paresthesia (one subject) and alteration of the sense of taste (two subjects). These adverse events resolved spontaneously. No adverse events were recorded following furosemide administration.

## Discussion

This randomized controlled investigation shows that acetazolamide reduces GFR in obese non diabetic subjects with glomerular hyperfiltration. A distally acting diuretic injected at an equipotent dose was used as control and showed no effect on GFR.

Previous studies showed that acetazolamide decreases GFR in animal models[40–43], in healthy humans[27–33] and in diabetic subjects[31,34]. The present investigation is the first to compare the effects of acetazolamide to those of an equipotent natriuretic agent and the first to investigate these effects in hyperfiltrating non-diabetic obese subjects.

Acetazolamide, a carbonic anhydrase inhibitor, acts on the proximal tubule by decreasing bicarbonate, sodium and chloride reabsorption. The increased solute delivery to the macula densa activates tubuloglomerular feedback leading to a reciprocal change in single nephron GFR, mostly resulting from changes in glomerular arterioles resistance[44]. The decrease in GFR has also been attributed to an increase in Bowman's space hydrostatic pressure secondary to inhibition of proximal reabsorption[45]. These changes result in a diminished transcapillary pressure gradient and a lowered single nephron GFR. GFR decreased following acetazolamide and remained unchanged following furosemide administration. Sodium balance, slightly negative following diuretic administration, was similar in both groups. Hence, changes in sodium balance did not account for the renal hemodynamic changes. Protein intake, a potential modulator of renal hemodynamics, was similar during the 24 hours preceding the two renal function studies.

The effects of acetazolamide on renal hemodynamics were compared to those of furosemide, a loop diuretic that increases natriuresis by inhibiting the sodium-potassium-2chloride co-transporter in the thick ascending limb of the loop of Henle. Acetazolamide and furosemide both increase solute distal delivery. However, the latter does not activate tubuloglomerular feedback[28,30,36–38] since it inhibits the sodium-potassium-2chloride co-transporter and therefore blocks solute transport into macula densa cells and the consequent decrease in glomerular filtration rate. A possible additional explanation for the lack of effect of furosemide on glomerular hyperfiltration may also be related to the cortical or global renal vasodilation demonstrated by some studies following intravenous administration of furosemide[46–50]. This effect may theoretically contribute to maintaining GFR. In the present study, renal vascular resistance did not change following furosemide. This finding does not support a role for a direct effect of furosemide on the renal vasculature in the settings of the present investigation, performed using low-dose furosemide in water-repleted subjects with high baseline renal plasma flow.

The dose of acetazolamide used in this investigation is similar to that used for clinical indications. Furosemide was administered at a low dose in order to match the natriuretic effect of acetazolamide. Preliminary studies showed that furosemide doses of 10 and 5 mg resulted in a more pronounced natriuretic effect than acetazolamide during the 60 min period following injection. We empirically determined that a 2 mg furosemide dose provides the sought natriuretic effect. A single study by Andreasen et al[51] reported the pharmacodynamics and kinetics of a 5 mg furosemide dose in young healthy adults, along with the effects of higher doses. As far as we know, no published data are available concerning the effects of lower doses. In this publication[51], the results for the 60 min period following injection of the 5 mg dose were provided as urine sodium excretion rate for the 0–15 min period and as fractional excretion of sodium (FENa) for the 15–30 and 30–60 min periods. By backcalculating natriuresis from FENa for the 15–30 and 30–60 min periods in that study, we obtain a urine sodium excretion rate of about 1.4 mmol/min. These findings are consistent with those of the present study, where the 2 mg dose induced about half the natriuresis generated by the 5 mg dose, i.e. 0.6 mmol/min. The authors noted that the median effective dose (ED50) of furosemide is "well below 5 mg", i.e. that the natriuretic response appears at doses much lower than 5 mg. These low doses are not used clinically in adults since the diuretic response wanes rapidly owing to the drug’s short half-life.

Baseline natriuresis was similar before acetazolamide and furosemide administration. Natriuresis increased likewise during the 60 min following administration of the two diuretics. The slightly negative sodium balance was similar in the two groups. During this time period, GFR decreased by 21% following acetazolamide, while it remained constant following furosemide. These findings suggest that the renal hemodynamic changes which occurred after acetazolamide were due to tubuloglomerular feedback activation and not to a systemic hemodynamic effect, nor to differences in natriuretic potency between furosemide and acetazolamide. Previous human studies on the effects of acetazolamide on renal hemodynamics were performed in diabetic and healthy subjects[27–34]. None of them included a control group that received a diuretic with similar effect on sodium excretion as the acetazolamide group. Fractional excretion of lithium was used in this study to evaluate renal sodium handling. Lithium is freely filtrated by the glomeruli, undergoes proximal tubular reabsorption by the same transport system as sodium, and is thereafter excreted without further significant reabsorption or secretion at more distal segments of the nephron[52]. Thus, fractional lithium excretion is a marker of proximal tubular sodium handling and its increase reflects a decrease in proximal sodium reabsorption. An exception to this rule is in the case of subjects under sodium restriction, where this relationship loses its validity[53]. This was not the case in the present investigation, where participants consumed a normal sodium diet. In this study, fractional lithium excretion increased by 61% following acetazolamide, reflecting a marked decrease in proximal sodium reabsorption. This marked increase is accounted for by the combined effect of a 30% increase in lithium clearance and the resulting 21% decrease in GFR. Fractional excretion of lithium increased also following furosemide, albeit more moderately. This probably reflects the fact that some lithium reabsorption occurs in the loop of henle[31,36,54,55]. To a lesser extent, a small inhibitory acute effect of furosemide on proximal tubular sodium reabsorption[55,56] might also account for the increase in lithium clearance. However, the evidence for this effect is conflicting[57]. The change in GFR following acetazolamide was inversely correlated with baseline GFR. Hannedouche et al31 previously showed that acetazolamide decreases GFR in diabetic and healthy subjects, the change in GFR being inversely correlated with baseline GFR. Similarly, Guidi et al[30] showed that the decrease in GFR following administration of acetazolamide to healthy individuals is more pronounced in subjects with high, as compared to those with low, baseline GFR. The present investigation confirms these findings in an obese population.

Most enrolled subjects had a urinary albumin excretion rate spanning within a range associated with increased risk for cardiovascular mortality[58]. The effects of acetazolamide and furosemide on albuminuria could not be estimated owing to the diluted state of the urine during the renal function studies, resulting in undetectable urinary albumin levels. Thus we were unable to determine whether acetazolamide affects this risk marker, in addition to its effects on glomerular hyperfiltration. The effects of acetazolamide on albumin excretion rate may not necessarily match those on glomerular filtration rate. It is of interest that baseline GFR predicted the effects of acetazolamide on glomerular hyperfiltration, while albuminuria did not. Thus, further investigations are required in order to assess the effects of acetazolamide on the latter.

Glomerular hyperfiltration is one of the factors responsible for the increased prevalence of chronic kidney disease in obese sujects[18–24,59]. Thus, decreasing glomerular pressure and single nephron GFR may protect the kidney from hyperfiltration-mediated injury. The available therapeutic tools aimed at decreasing glomerular filtration pressure are antihypertensive treatment, inhibition of the renin angiotensin, weight loss[7,17] and low salt diet[13]. Ogna et al[60] recently showed in a population-based survey that overweight and obesity, and to a lesser extent salt intake, are independent predictors of glomerular hyperfiltration in the general population. These findings raise the hypothesis that decreasing salt intake may attenuate hyperfiltration. Krikken et al[13], measuring glomerular function in healthy lean and overweight subjects on a low- and a high-sodium diet, demonstrated that the change from low- to high-sodium diet led to an increase in filtration fraction in the overweight group, while no change was found in the lean group, suggesting that a low salt diet may attenuate glomerular hyperfiltration in subjects with increased adiposity. It should, however, be noted that this study was performed in normotensive subjects and that the glomerular hemodynamic response to a change in salt intake may differ in hypertensive and in normotensive subjects[61], irrespective of the obesity status.

Abating hyperfiltration through tubuloglomerular feedback activation provides a new approach to this issue, by directly acting on one of the mechanisms causing obesity-induced hyperfiltration. Non diabetic obese subjects have increased fractional sodium reabsorption in the proximal tubule[14,62,63]. This abnormal tubular sodium handling also occurs in diabetic murine models and diabetic humans[64–66]. Vallon et al[66,67] presented data obtained in a murine diabetic model suggesting that a primary proximal tubular factor leads to enhanced proximal tubular reabsorption, decreased solute delivery to the macula densa and deactivation of the tubuloglomerular feedback, with consequent glomerular hyperfiltration. The results of the present study are compatible with this concept, although do not disprove the involvement of primary vascular factors[24] in the pathogenesis of glomerular hyperfiltration. The present study is the first to investigate the effects of tubuloglomerular feedback manipulation in obese non diabetic subjects. This effect, which was previously demonstrated in diabetic and healthy subjects[27–34], is not specific to the obese population.

The short term design of the present study does not allow inferring about the effectiveness of acetazolamide in the long-term in obese subjects. Recently, Cherney et al[35] studied in type 1 diabetic subjects, the effects of an eight-week treatment with empagliflozin, a sodium glucose co-transporter 2 inhibitor that decreases proximal tubular glucose and sodium reabsorption and thus increases solute delivery to the macula densa. The authors showed that this treatment leads to a decrease in GFR in type 1 diabetic subjects with glomerular hyperfiltration. This result suggests that in this population, attenuation of glomerular hyperfiltration by tubuloglomerular feedback manipulation using a sodium glucose co-transporter 2 inhibitor is feasible during a 2-month period. Is the hypofiltrating effect of acetazolamide maintained in the long-term? Acetazolamide is rarely used as a diuretic since its long-term natriuretic effect is modest. The main factors accounting for this are the increased reabsorption of the excess solute and fluid delivered distally[68], the decrease in GFR, consequent to tubuloglomerular feedback activation, leading to a decline in the filtered sodium load[45,68] and the decrease in plasma bicarbonate and consequent reduction in glomerular filtrate bicarbonate concentration to a level lower than the maximal reabsorptive capacity of the nephron, thus abating distal delivery of sodium and bicarbonate. Does the short-term diuretic effect of acetazolamide imply an ephemeral effect on GFR? Lorenz et al68 studied knockout mice for the Na/H exchanger isoform 3 which mediates 60% of salt reabsorption in the proximal tubule. Single nephron GFR of these knockout mice was lowered owing to tubuloglomerular feedback activation. This long-term hypofiltrating effect in animals with Na/H exchanger isoform 3 deficiency suggests that chronic carbohydrase inhibition would similarly result in long-term glomerular hypofiltration.

Skott et al[69] showed that a 3-day treatment course of oral acetazolamide decreases GFR in diabetic patients with mild chronic renal insufficiency and in healthy subjects. The 24-hour urine sodium excretion, which was markedly increased at Day 1 of the treatment, as compared to the pre-treatment level, decreased at Day 3 to a level similar to the pre-treatment level. This suggests that the hypofiltrating effect of acetazolamide still persists at a time when the diuretic effect has vanished. However, no conclusive data are available regarding its long-term effect on glomerular filtration. Therefore, the long-term effects of natriuretic agents acting on the proximal tubule, such as carbohydrase inhibitors and sodium glucose co-transporter 2 inhibitors, should be investigated in hyperfiltrating subjects at risk for advanced chronic kidney disease. Of note, if the long-term hypofiltrating effect of acetazolamide was demonstrated, its side effects[70]— nephrolithiasis, lethargy, paresthesia and mainly the ineluctable metabolic acidosis—may preclude its use as a chronic treatment for glomerular hyperfiltration.

The strengths of the present study are its randomized double blind design, the use of a diuretic injected at equipotent dose, the exclusion of diabetic subjects and attainment of a similar sodium balance in the acetazolamide and furosemide groups. Its limitations are the small number of subjects involved and its short term design. The cross over design of the study enabled us to demonstrate the effect of the study drug despite a small number of participants.

In summary, manipulating tubuloglomerular feedback with acetazolamide is effective in acutely abating glomerular hyperfiltration in obese non diabetic subjects.

## Supporting Information

S1 CONSORT Checklist (PDF)

S1 Dataset (XLS)

S1 Study Protocol English (PDF)

S1 Study Protocol Hebrew (PDF)
