# Supplementary material for: Effect of Acetazolamide on Obesity-Induced Glomerular Hyperfiltration: A Randomized Controlled Trial
Source: PLoS One. 2015 Sep 14;10(9):e0137163. doi: 10.1371/journal.pone.0137163 (PMC4569381; doi:10.1371/journal.pone.0137163)
Supplement: S1 Study Protocol English — (PDF) [file pone.0137163.s003.pdf]

Effect of Acetazolamide and Furosemide on obesity-induced glomerular hyperfiltration

Boris Zingerman, MD, Avry Chagnac, MD, Uzi Gafter, MD, PhD

Department of Nephrology and Hypertension

Rabin Medical Center – Hasharon Hospital

V 3. December 2010

BACKGROUND

METHODS

<u>Inclusion criteria:</u>

15 obese men (BMI>30), aged 18 to 55, with glomerular hyperfiltration (creatinine clearance>130 ml/min) and 10 normal body weight men (BMI<25), aged 18 to 55, will be included in the study.

<u>Exclusion criteria:</u>

Any of the following conditions:

- Heart failure, CKD, COPD
- Known allergy to furosemide, acetazolamide, inulin or amino-hippurate
- Pharmacologic treatment for hypertension, cardiac disease, diabetes mellitus
- Treatment with corticosteroids, antiepileptics or NSAID

<u>Methods:</u>

A 24-hour urine collection will be performed during the week prior to the renal function test studies for assessment of sodium intake.

<u>Obese subjects</u>: A randomized double-blind crossover controlled design will be used. Two renal function studies will be performed: one before and after intravenous furosemide and the second before and after intravenous acetazolamide. Subjects will receive 300 mg of lithium carbonate at 22.00 the day before the renal function tests. Renal function tests will start at 08.00 a.m.

after a 10-hour fast. Intravenous catheters will be placed in each upper limb for infusion of clearance markers and blood sampling. After blood sampling for urea, creatinine, proteins, glucose, electrolytes, blood gases, insulin, renin, aldosterone, Hba1c, CBC. A priming dose of inulin (40 mg/kg) and p-aminohippuric acid (4 mg/kg) will be administered p.o. and a 10 ml/ kg BW water load will be given. Thereafter, inulin 25% and p-aminohippuric acid 20% will be infused continuously at a dose of 8 cc/hr and 4 cc/hr, respectively. After the first 60 minutes, 8 accurately timed urine collections of 30 minutes will be obtained by spontaneous voiding. Peripheral venous blood will be drawn to bracket each urine collection. Arterial pressure will be measured by a trained observer, after 30 minutes of rest in the supine position, using an electronic oscillometric blood pressure measuring device (Datascope, Accutorr). The cuff will be appropriately sized to the diameter of the arm and the arm positioned at the heart level. At least 8 measurements will be performed during the study, each measurement being the mean of 3 readings. After the first 4 timed urine collections, participants will receive intravenous furosemide 2 mg within 5 min or intravenous acetazolamide 5 mg/kg within 5 min. Four other times urine collections will be performed thereafter. Subjects will be randomized to receive during the first study either furosemide or acetazolamide. The second study will be performed one to two weeks after the first study, using the drug that had not administrated during the first study.

Subjects with normal body weight: will undergo measurement of renal function without administration of diuretics (one renal function study, same protocol like obese subjects, with 4 urine collections only).

Laboratory procedures: Plasma and urinary concentrations of inulin and p-aminohippuric acid will be analyzed by colorimetric methods (22,23). Lithium in serum and urine will be measured using the ICP-OES (Inductively Coupled Plasma Optical Emission Spectrometer) method. Urine microalbumin will be determined by competitive chemiluminescent enzyme immunoassay (Imulite, DPC, Los Angeles, CA, USA).

Calculations: GFR will be determined from the average value for the timed inulin clearances, and renal plasma flow (RPF) – from the average value for the timed p-aminohippurate clearances. The fractional excretion of lithium (FE Li) will be calculated as lithium clearance / GFR, using two timed urine collections. FE Li will be determined as the average value for these two measurements

Statistical Analysis: The significance of differences between groups will be evaluated by paired and unpaired two-tailed Student's t-test. The Student's *t*-test will be applied to non-normally distributed data (albumin excretion rate and fractional lithium excretion) after log transformation.
