# Supplementary material for: Effect of Acetazolamide on Obesity-Induced Glomerular Hyperfiltration: A Randomized Controlled Trial
Source: PLoS One. 2015 Sep 14;10(9):e0137163. doi: 10.1371/journal.pone.0137163 (PMC4569381; doi:10.1371/journal.pone.0137163)
Supplement: S1 Study Protocol Hebrew — (PDF) [file pone.0137163.s004.pdf]

השפעת Acetazolamide וFurosemide על ההיפרפילטרציה הגלומרולרית באנשים עם השמנת יתר

ד"ר בוריס זינגרמן, פרופ' אברי שניאק, פרופ' עוזי גפטר

המערך לנפרולוגיה ויתר לחץ דם
מרכז רפואי רבין - קמפוס גולדה

Effect of Acetazolamide and Furosemide on obesity-induced glomerular hyperfiltration

Boris Zingerman,MD, Avry Chagnac,MD, Uzi Gafter, MD, PhD

Department of Nephrology and Hypertension
Rabin Medical Center – Golda Campus

גירסה 3 - דצמבר 2010

רקע

...

מטרת המחקר

מטרת המחקר הינה לבדוק האם acetazolamide במתן תוך ורידי חד פעמי ממתן את ההיפרפילטרציה הגלומרולרית משנית להשמנת יתר ואם השפעה זו משנית להשפעה על ה tubulo-glomerular feed-back. לצורך כך, נשווה את השפעת acetazolamide להשפעת משתן אחר - furosemide- אשר אינו משפיע על ה- tubulo-glomerular feed-back.
המחקר הינו אקראי מבוקר כפול סמיות.

פרוטוקול

אוכלוסית הנבדקים

תנאי הכללה:

15 גברים ונשים עם השמנת יתר (BMI מעל 30) ו10 גברים ונשים עם משקל גוף תקין (BMI פחות מ 25) בגילאים 18 עד 55 ישתתפו במחקר.
הנבדקים עם השמנת יתר יעברו לפני ההכללה במחקר בדיקת פינוי קראטינין (בדיקת קראטינין בדם ואיסוף שתן של 24 שעות לבדיקת קראטינין). יכללו נבדקים עם פינוי קראטינין מעל 130 סמ"ק לדקה. הנבדקים ללא השמנת יתר יעברו לפני ההכללה במחקר בדיקת קראטינין בדם. יכללו נבדקים ללא השמנת יתר עם קראטינין בנסיוב תקין.

תנאי אי-הכללה:

חולים הסובלים מאי ספיקה כליתית., אי ספיקת לב, מחלה ריאתית חסימתית כרונית, הפרעה באלקטרוליטים (סודיום, אשלגן, ביקרבונט) ואלרגיה ידועה ל-furosemide, inulin, aminohipurate, או acetazolamide לא יכללו בניסוי. כמו כן, לא ישתתפו במחקר נשים בהריון, חולים המקבלים טיפול תרופתי נגד סכרת, יתר לחץ דם, מחלות לב או טיפול באספירין במינון גבוה (מעל 100 מ"ג ליום) ותרופות נגד אפילפסיה.

שיטות

לפני בדיקת תפקודי הכליה יבוצעו איסוף שתן של 24 שעות לבדיקת הפרשת אוראה, קראטינין, סודיום ואלבומין בשתן. בנוסף יבצעו בדיקות קראטינין, אורה, נתרן, אשלגן, סוכר בדם.

בדיקת תפקודי כליות:

קבוצת השמנת יתר: כל נבדק מקבוצת השמנת יתר יעבור פעמיים בדיקת תפקודי כליות במרווח של שבוע עד שבועיים. האחת לפני ואחרי מתן iv furosemide והשנייה לפני ואחרי

מתן iv acetazolamide. יום לפני בדיקה תפקודי הכליות, הנבדקים יאספו שתן למשך 24 שעות לבדיקת הפרשת אוראה, קראטינין, סודיום ואלבומין בשתן.

הסדר ייקבע בצורה אקראית.

הנבדקים ייטלו 300 מ"ג lithium carbonate בדרך פומית 10 שעות לפני בדיקות תפקודי הכליות (טבליה אחת של licarbium). הפרשת הליתיום בשתן תשמש מדד לספיגת הסודיום בטובול הפרוקסימלי. בדיקת תפקודי כליות תתחיל ב-8:00 בבוקר בצום. הנבדקים יקבלו דרך צנטר בווריד היקפי מנת העמסה של inulin 25% במינון של 40 מ"ג לק"ג משקל ו- amino hippurate sodium 20% במינון 4 מ"ג לק"ג משקל, ולאחר מכן יינתן עירוי מתמשך של inulin 25% במינון של 8 סמ"ק לשעה ו- amino hippurate sodium 20% במינון של 4 סמ"ק לשעה. הנבדקים ישתו מים בכמות של 10 סמ"ק לק"ג משקל גוף, ולאחר מכן שתית כמות מים זהה לכמות אשר תוטל לאחר כל הטלת שתן.לאחר שעה של equilibration יתבצעו 8 איסופי שתן של 30 דקות. לאחר 4 איסופים הראשונים, הנבדקים יקבלו iv furosemide 2 mg תוך 5 דקות או iv acetazolamide 5 mg/kg BW תוך 5 דקות.

תילקחנה דגימות דם לפני ובסוף כל איסוף שתן. בדיקות הדם והשתן תבדקנה לבדיקת אינולין, אמינוהיפורט, סודיום, אשלגן, , pHביקרבונט, כלוריד, ליתיום ואלבומין, ספירת דם . . בנוסף, ריכוז CRP, פרופיל שומני, גלוקוז,HbA1c, ייבדקו בבדיקת דם לפני האיסוף שתן הראשון. בדיקות renin וaldosterone- ייבדקו בדגימות דם אשר תילקחנה לפני איסוף השתן הראשון ואחרי איסוף שתן הרביעי, החמישי והשביעי. לחץ הדם ייבדק בסיום כל איסוף שתן (8 פעמים).

הנבדקים שקיבלו acetazolamide בבדיקה הראשונה יקבלו furosemide בפעם השנייה ולחילופין. המחקר הינו אקראי מבוקר כפול סמיות.

קבוצת הנבדקים במשקל גוף תקין: כל נבדק מקבוצת משקל גוף תקין יעבור בדיקת תפקודי כליות בסיסית פעם אחת בלבד: הפרוטוקול זהה לפרוטוקול של הקבוצה עם השמנת יתר ללא מתן acetazolamide או furosemide .

לפני בדיקת תפקודי כליות הנבדקים יקבלו תוספת טבליות מלח למשך שבוע. 3 ו6 ימים לאחר קבלת מלח יבוצעו 2 איסופי שתן 24 שעות לבדיקת הפרשת סודיום וקראטינין בשתן.

יינתן תשלום למשתתפים.

ההשגחה הרפואית תיעשה במחלקת נפרולוגיה. על יד המטופל ימצא ציוד החייאה ואיש צוות רפואי לאורך כל משך הניסוי.

<u>עיבוד הנתונים</u>

יחושבו:

GFR ייחשב כ-inulin clearance.

RPF ייחשב כ-amino-hippurate clearance

GFR/RPF = filtration fraction

fractional lithium clearance (clearance lithium/GFR)

fractional albumin clearance (clearance albumin/GFR)

ההשוואה הסטיסטית בין המשתנים השונים לפני ואחרי הטיפול תעשה בעזרת Student's paired t-test. משתנים עם פיזור לא נורמלי יעברו log-transformation לפני ישום ה t-test.
